# Supplementary figures and images for: A super-resolution network using channel attention retention for pathology images
Source: PeerJ Comput Sci. 2023 Jan 17;9:e1196. doi: 10.7717/peerj-cs.1196 (PMC10280234; doi:10.7717/peerj-cs.1196)

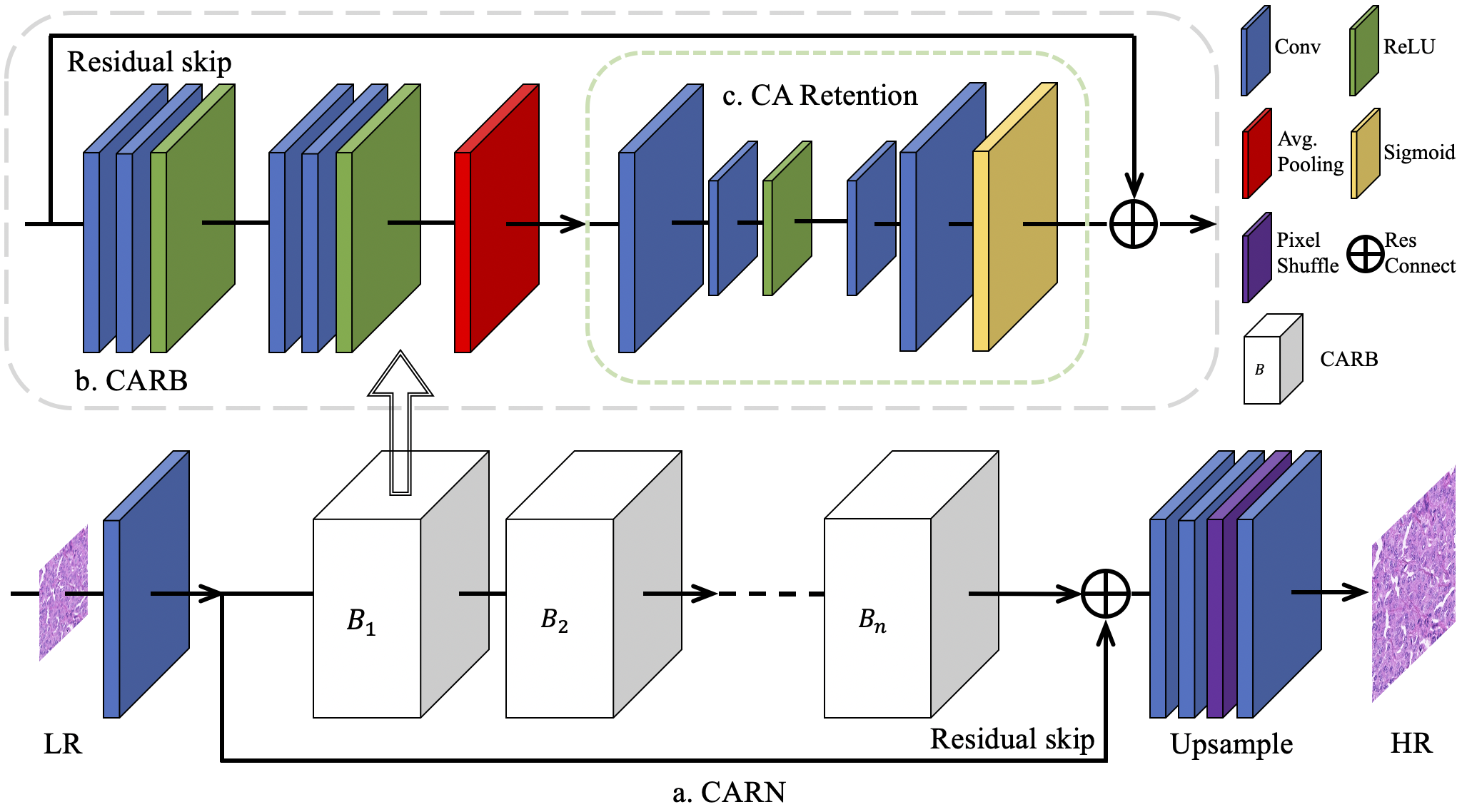

Supplement: Supplemental Information 1 — From GitHub: https://github.com/MoyangSensei/CARN-Pytorch. The code and dataset bcSR are described in README.md. Write with Python. [file peerj-cs-09-1196-s001.zip › CARN-Pytorch-main/fig/11.png]

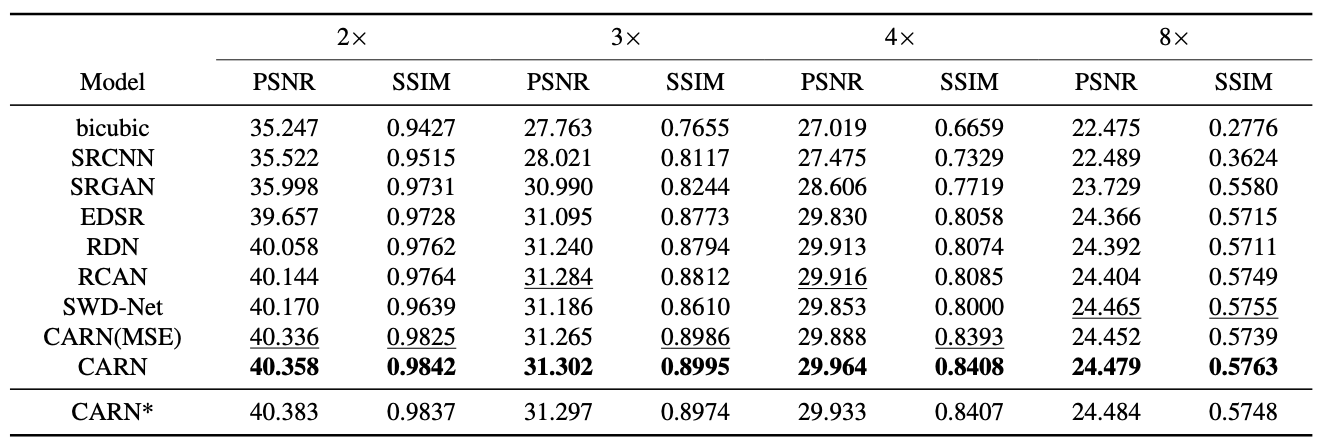

Supplement: Supplemental Information 1 — From GitHub: https://github.com/MoyangSensei/CARN-Pytorch. The code and dataset bcSR are described in README.md. Write with Python. [file peerj-cs-09-1196-s001.zip › CARN-Pytorch-main/fig/22.png]

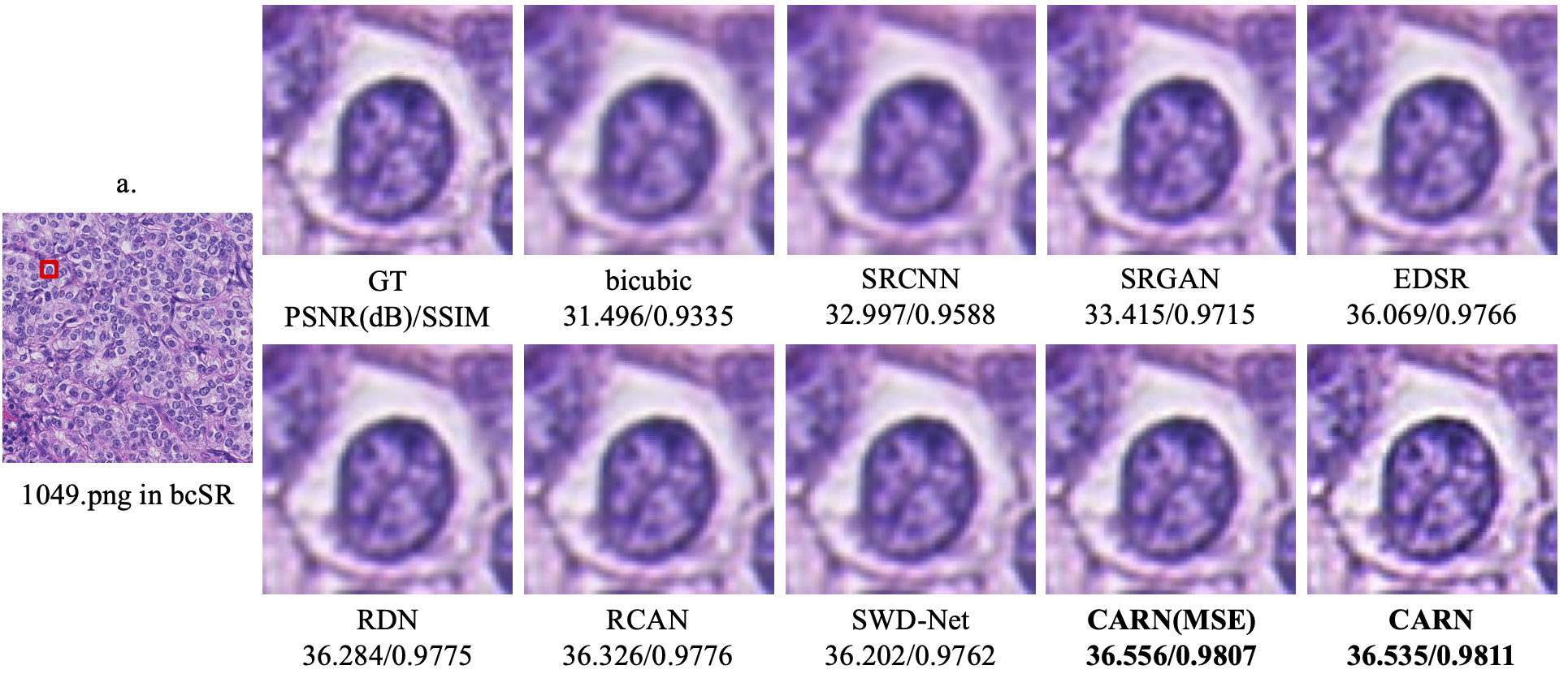

Supplement: Supplemental Information 1 — From GitHub: https://github.com/MoyangSensei/CARN-Pytorch. The code and dataset bcSR are described in README.md. Write with Python. [file peerj-cs-09-1196-s001.zip › CARN-Pytorch-main/fig/33.png]

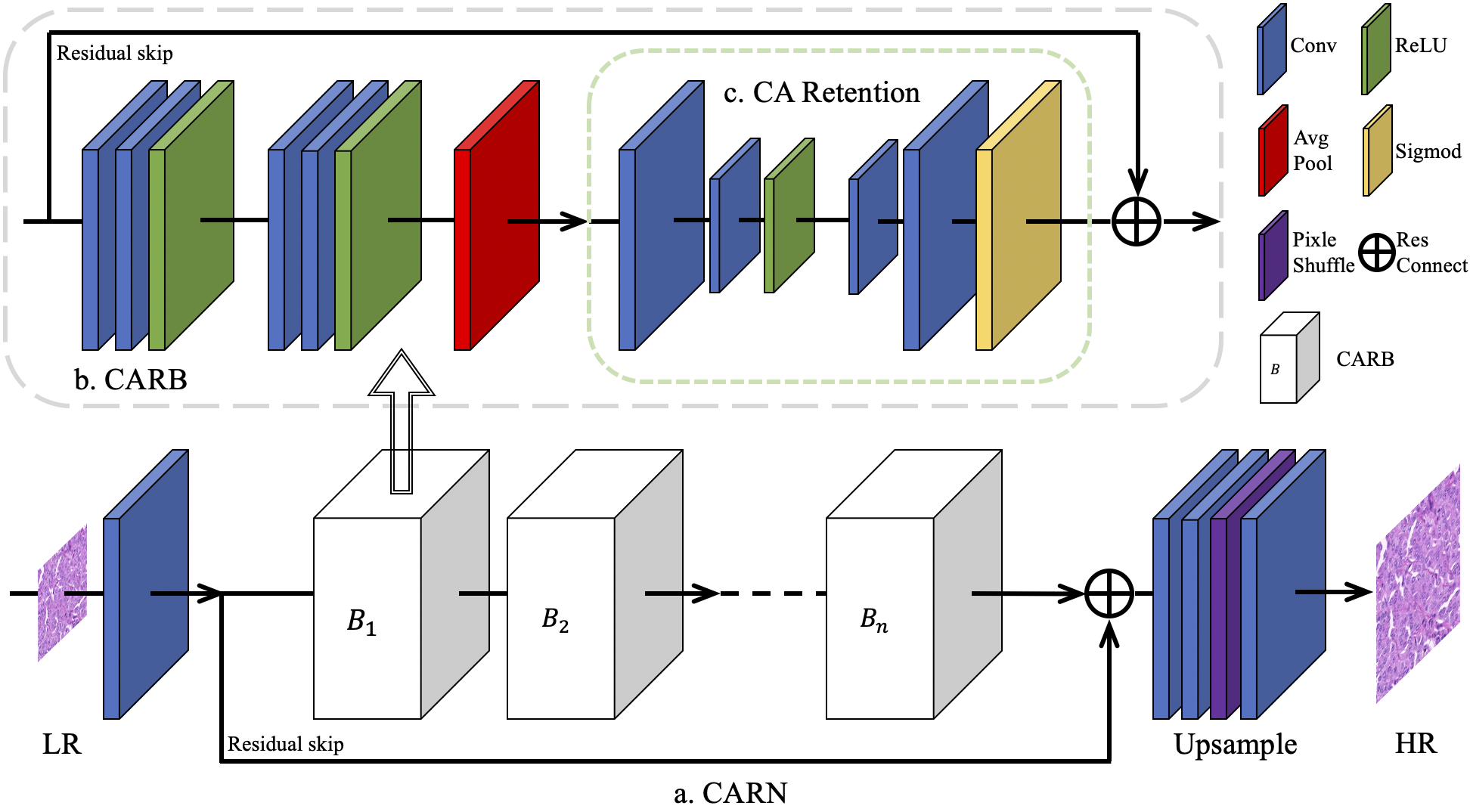

Supplement: Supplemental Information 5 — From GitHub: https://github.com/MoyangSensei/CARN-Pytorch. The code and dataset bcSR are described in README.md. Write with Python. [file peerj-cs-09-1196-s005.zip › CARN-Pytorch-main/fig/1.png]

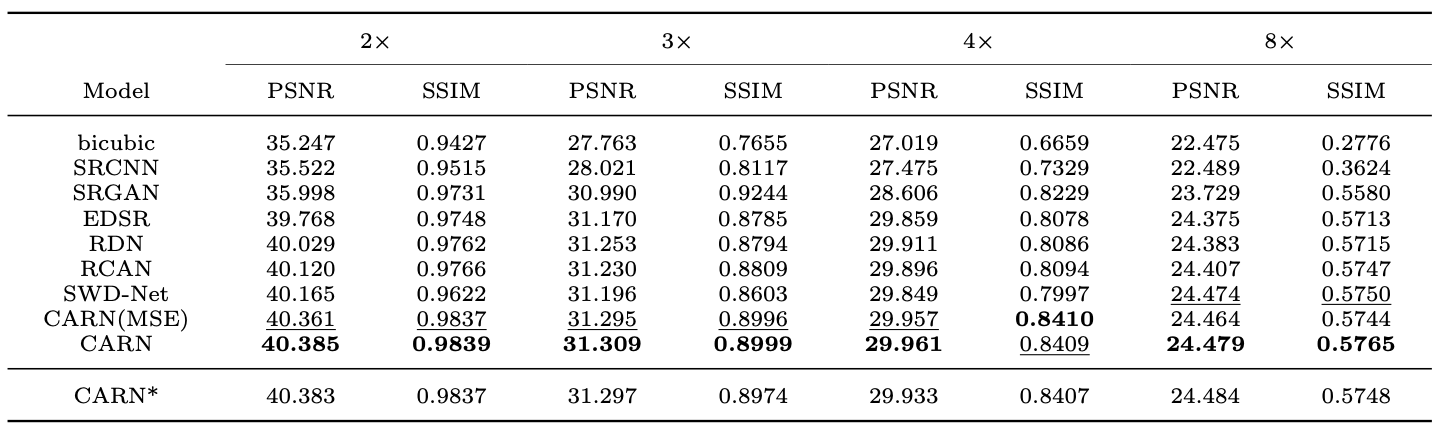

Supplement: Supplemental Information 5 — From GitHub: https://github.com/MoyangSensei/CARN-Pytorch. The code and dataset bcSR are described in README.md. Write with Python. [file peerj-cs-09-1196-s005.zip › CARN-Pytorch-main/fig/2.png]
